# Lineage-aware comparison of extended-spectrum β-lactamase-producing Escherichia coli from unweaned dairy calves and human references reveals host-structured plasmidomes and co-selection

**DOI:** 10.1099/mgen.0.001783

**Published:** 2026-07-17

**Authors:** Mykhailo Savin, Jason Jeremia Hayer, Nico T. Mutters, Tim Erler, Sophie Simon, Lea Griesdorn, Julia Steinhoff-Wagner, Jens A. Hammerl, Céline Heinemann, Alexander J. Probst

**Affiliations:** 1 Institute for Hygiene and Public Health, University Hospital Bonn, University of Bonn, Bonn, Germany; 2 Educational and Research Centre for Animal Husbandry, Hofgut Neumuehle, Muenchweiler an der Alsenz, Germany; 3 Environmental Metagenomics, Research Center One Health Ruhr of the University Alliance Ruhr, University of Duisburg-Essen, Essen, Germany; 4 TUM School of Life Sciences, Technical University of Munich, Freising-Weihenstephan, Germany; 5 Department Biological Safety, German Federal Institute for Risk Assessment, Berlin, Germany; 6 Institute of Animal Sciences, University of Bonn, Bonn, Germany

**Keywords:** antimicrobial resistance, calves, *Escherichia coli*, metal/biocide resistance, mobile genetic elements, plasmid architecture

## Abstract

Antimicrobial resistance in *Escherichia coli* is shaped not only by resistance genes themselves but also by their chromosomal or plasmid localization and co-occurrence with biocide/metal resistance genes (BMRGs), virulence-associated genes and mobile genetic elements. We applied chromosome- and plasmid-resolved genomics to 109 extended-spectrum *β*-lactamase-producing *E. coli* isolates from unweaned dairy calves (*n*=484) in Germany and compared them with 479 human-associated reference genomes. Calf isolates were polyclonal and dominated by phylogroups A and B1. Resistance was predominantly plasmid-borne: 41% of isolates carried antibiotic resistance genes (ARGs) exclusively on plasmids, whereas only 4.6% carried ARGs exclusively on chromosomes. The chromosomal-versus-plasmid distribution of acquired ARGs differed significantly across phylogroups (*P*<0.05) and sequence types (all *P*<0.01). Conjugative plasmids accounted for 94.6% of plasmid-borne ARG occurrences and carried significantly more ARGs than mobilizable plasmids (*P*=3.66×10^−42^). ARG and BMRG counts were strongly correlated at the plasmid level (ρ=0.574, *P*=8.0×10^−41^), and class 1 integrons marked enriched multidrug plasmids with increased ARGs (*P*=6.22×10^−34^) and BMRGs (*P*=3.00×10^−29^). At the isolate level, calf isolates carried more acquired ARGs in unadjusted comparisons, but this host-associated difference was largely explained by population structure. At the plasmid level, however, host-associated differences persisted after adjustment: human plasmids carried more ARGs (IRR 1.66, *P*=0.0017) and showed a strong host×mobility interaction (IRR 4.61, *P*=4.9×10–8), stronger ARG–BMRG coupling and a higher prevalence of integrons. These findings show that antimicrobial resistance ecology in *E. coli* is shaped not only by which resistance genes are present, but by where they are located, what they are linked to and how readily their genomic carriers can disseminate.

**Impact Statement**

Extended-spectrum *β*-lactamase (ESBL)-producing *Escherichia coli* are important One Health indicators of antimicrobial resistance, yet most genomic studies focus primarily on resistance gene content rather than the chromosomal or plasmid context in which these genes are embedded. Here, we combine chromosome- and plasmid-resolved genomics with lineage-aware comparative analyses to examine ESBL-producing *E. coli* from healthy unweaned dairy calves and to place these findings in the context of a curated human-associated reference collection processed through the same workflow. We show that resistance in calf isolates is predominantly plasmid-borne and concentrated in a limited number of conjugative, IncF-enriched plasmid backbones, with class 1 integrons and linked biocide/metal resistance genes marking multidrug platforms with high co-selection potential. Importantly, many apparent host-associated differences were attenuated after controlling for population structure, whereas plasmid-level differences persisted, indicating that resistance divergence between hosts is driven less by gene content alone than by plasmidome organization, mobility and co-localization patterns. This shifts antimicrobial resistance surveillance from simple resistance gene detection towards evaluating genomic vehicles, co-selective cargo and the potential for persistence and cross-host dissemination.

## Data Summary

Genome assemblies of the *Escherichia coli* isolates were deposited in GenBank (NCBI) under BioProject PRJNA1440894.

## Introduction

Genomic studies of antimicrobial resistance (AMR) often emphasize resistance gene content and lineage composition. However, resistance development is shaped not only by gene presence, but also by how these genes are embedded within the bacterial genome [1,3]. As antibiotic, biocide and metal residues accumulate across environmental compartments, the genomic context of antibiotic resistance genes (ARGs) becomes increasingly relevant [4,5]. Whether ARGs are chromosomally encoded or plasmid-borne, enriched in conjugative versus non-conjugative backbones and co-localized with biocide and metal resistance genes (BMRGs) or virulence-associated loci can strongly influence persistence, dissemination and co-selection, while chromosomal integration or stable plasmid maintenance may make loss of these determinants unlikely. Understanding AMR ecology and exchange therefore requires a structural view of resistance across chromosomes and plasmids [1,6, 7]. Here, we distinguish acquired ARGs from chromosomal resistance-associated mutations. While the former represent horizontally acquired determinants frequently linked to mobile genetic elements, the latter result from nucleotide changes in endogenous chromosomal genes. This distinction is important because both mechanisms differ in mobility, stability and dissemination potential [8,9].

*Escherichia coli* is a key model organism for genomic AMR studies and One Health surveillance, spanning human, animal and environmental compartments [10,12]. Extended-spectrum *β*-lactamase (ESBL)-producing *E. coli* are particularly relevant because they combine ecological versatility with a high capacity for horizontal gene transfer, enabling persistence and dissemination of resistance across hosts and settings [10,12].

Livestock-associated *E. coli*, including calf-associated populations, provide a relevant model for studying how AMR persists, disseminates and is co-selected under farm-associated selective pressures [13,14]. Calf production systems involve dense host contact, frequent microbial exchange and multiple selective pressures, including antimicrobials, disinfectants and metal-associated stressors [13,15]. These conditions may favour the maintenance of multidrug resistance plasmids and linked co-selection patterns [14,15]. Yet several key questions remain unresolved, including the extent to which resistance is concentrated in specific plasmid backbones, how strongly plasmid-mediated resistance is shaped by lineage background and whether co-selection signatures are broadly distributed or confined to recurrent plasmid hubs [4,16].

Cross-host comparisons with human-associated *E. coli* could provide important context, but they are often difficult to interpret. Publicly accessible collections of human-associated genomes are typically heterogeneous in sampling strategy, sequencing approach and clinical enrichment and may be biassed towards specific pathotypes or high-risk clones [17,18]. As a result, apparent host-associated differences can reflect lineage composition or technical heterogeneity rather than biological differences in resistance [2,3, 19].

To address these challenges, we combined chromosome- and plasmid-resolved genomics with phylogroup- and ST-aware comparative analyses to assess not only which resistance genes were present, but also where they were located and how their distribution was shaped by lineage and host context. We analysed ESBL-producing *E. coli* from clinically healthy unweaned calves and placed their resistance patterns and plasmidome organization in the context of a curated set of complete human-associated reference genomes processed through the same pipeline. Importantly, the human reference dataset was not preselected for ESBL phenotype, allowing host-context comparisons that are less constrained by phenotype-specific sampling.

Within this framework, we pursued three interlinked aims. First, we defined the population structure of ESBL-producing calf *E. coli* (*n*=109) by Clermont phylogroup classification and MLST and examined how acquired resistance genes were distributed between chromosomes and plasmids. Second, we characterized the calf plasmidome with respect to replicon types, predicted mobility classes, plasmid clusters and integron content to identify recurrent backbones and mechanisms that concentrate acquired resistance and enable co-selection. Third, we embedded the calf plasmidome in a broader host context by comparing isolates and plasmids with a curated, non-enriched, worldwide reference collection of complete human-associated *E. coli* genomes (*n*=479) processed with the same workflow. Comparative analyses were explicitly phylogroup- and ST-aware to disentangle host-associated patterns from lineage structure and to assess how lineage background, plasmid mobility, backbone identity and integron content jointly shape resistance, co-selection and accessory gene organization across host-associated *E. coli* populations.

## Methods

### Sampling and isolation of ESBL-producing *E. coli*

Rectal and nasal swabs (eSwab, liquid Amies; Copan, Brescia, Italy) were collected from 484 unweaned dairy calves on 23 farms in Rhineland-Palatinate and North Rhine-Westphalia, Germany, between October and December 2019 as part of a predefined project sampling schedule. Calves were predominantly Holstein–Friesian, with 17 Jersey calves included. The study population consisted mainly of females (392/484, 81.0%), and most animals were group-housed at sampling (351/484, 72.5%). The mean age was 45.6±30.3 days.

Swabs were transported under cooled conditions and processed within 24 h. Aliquots were enriched in Mossel broth (24 h, 41 °C, 170 r.p.m., aerobic conditions), plated on CHROMagar ESBL (Mast Group, Reinfeld, Germany) and incubated for 24 h at 41 °C. Presumptive ESBL-producing *E. coli* colonies were subcultured on Columbia blood agar, identified by MALDI-TOF MS (Bruker Daltonics GmbH and Co. KG, Germany) and stored at −80 °C until further analysis.

### Antimicrobial susceptibility testing

MICs were determined by broth microdilution using the Micronaut-S MDR MRGN Screening system (Bruker Daltonics GmbH and Co. KG, Germany) according to the CLSI broth microdilution standard M07-A10 [20]. Results were interpreted using EUCAST clinical breakpoints (v15.0, 2025), reflecting the European/German clinical interpretation framework.

### DNA extraction, sequencing and assembly

Genomic DNA was extracted from overnight cultures of all 109 ESBL-producing *E. coli* isolates using the MasterPure Complete DNA and RNA Purification Kit (Lucigen, Biosearch Technologies, Novato, CA, USA). DNA quantity and quality were assessed using Qubit 1× dsDNA HS Assay Kit (Thermo Fisher Scientific, Waltham, MA, USA), NanoDrop ONE spectrophotometer (Thermo Scientific, Waltham, MA, USA) and TapeStation 4200 system (Agilent Technologies, Santa Clara, CA, USA). Libraries were prepared with the ONT Rapid Barcoding Kit (SQK-RBK114.96; Oxford Nanopore Technologies, Oxford, OX4 4DQ, UK) and sequenced on a PromethION P2 Solo using R10.4.1 flow cells, achieving a median coverage of 60× per genome. Basecalling was performed with Dorado v0.9.6 (super-accurate; v5.0.0 SUP model; https://github.com/nanoporetech/dorado), reads were filtered with Filtlong v0.2.1 (Q≥20; length ≥1,000 bp; https://github.com/rrwick/Filtlong), and assemblies were generated with Flye v2.9.6 [21], using metaFlye where appropriate [22].

### Human reference dataset

For host-context comparisons, 479 complete human-associated *E. coli* genomes were retrieved from NCBI RefSeq. Genomes were included if they fulfilled the following criteria: species annotation as *E. coli*, BioSample host metadata annotated as *Homo sapiens*, RefSeq assembly status as complete genome, assembly generation from ONT-only or hybrid ONT/short-read sequencing data, fewer than 10 assembled contigs, estimated completeness ≥99% and estimated contamination <1%. The dataset included isolates from clinical and colonization/screening specimens collected worldwide between 1954 and 2024 (predominantly 2018–2023). Importantly, genomes were not preselected for ESBL phenotype or resistance status, allowing comparison with a broad human-associated reference population rather than an ESBL-enriched dataset. Accession numbers and associated metadata are provided in Table S2, available in the online Supplementary Material. Assemblies were generated either from ONT-only data (*n*=175) or hybrid ONT/short-read approaches (*n*=304). All genomes were processed through the same pipeline as the calf dataset.

### Comparative genomics and feature calling

Chromosomal and plasmid fractions were delineated using MOB-suite (v3.1.9), which was also used to assign plasmid replicons, mobility class and plasmid clusters via MOB-typer [23,24]. ARGs, *E. coli* virulence factors (VFs) and BMRGs were identified using Abricate (v1.2.0; https://github.com/tseemann/abricate) with the ResFinder [25], ecoli_vf [26] and BacMet databases, respectively. Clermont phylogroup classification and MLST were obtained using the Bakcharak pipeline [27]. Integrons were detected with IntegronFinder (v2) [28].

### Statistical analyses

All analyses were performed in R [29] using binary presence–absence matrices derived from Abricate hits with a percentage alignment (PA) ≥90% calling criterion. Gene counts were calculated as the number of PA-positive genes per unit of analysis. Analyses were conducted at the isolate level (calf, *n*=109; human, *n*=479) and plasmid level (calf, *n*=450; human, *n*=1,163). Continuous variables were summarized as medians and interquartile ranges (IQRs) and compared using Kruskal–Wallis tests with Wilcoxon rank-sum post-hoc tests. Multiple testing was controlled using the Benjamini–Hochberg (BH) adjustment.

To account for population structure, host comparisons were repeated in plasmid-positive isolates using sequence type (ST)-matched paired Wilcoxon tests on per-ST median plasmid ARG counts and negative binomial models, including host plus ST or host×phylogroup terms. At the plasmid level, ARG counts were modelled by negative binomial regression with host, mobility class, phylogroup and host×mobility interaction; logistic regression for ARG presence/absence served as sensitivity analysis. Associations between quantitative features were assessed using two-sided Spearman correlations, including phylogroup-stratified and conjugative-plasmid-restricted analyses where indicated. Binary enrichment and gene-level co-occurrence were tested using Fisher’s exact tests with odds ratios (ORs) and 95% confidence intervals; gene-pair screening used BH-adjusted q-values. Categorical distributions were compared using χ² tests. Statistical significance was defined as two-sided *P*<0.05 or q<0.05

## Results

A total of 109 presumptive ESBL-producing *E. coli* isolates were recovered from 77 of 484 calves (15.9%), originating from 19 of 23 farms (82.6%). Most isolates were obtained from rectal swabs (*n*=88), whereas 21 isolates were recovered from nasal swabs; five calves yielded presumptive ESBL-producing *E. coli* from both rectal and nasal swabs.

### Phenotypic resistance profiles

All calf *E. coli* isolates were resistant to cefotaxime (109/109, 100%), as expected given their initial selection on ESBL-selective media. High resistance frequencies were also observed for piperacillin (107/109, 98.2%). Resistance to trimethoprim–sulfamethoxazole and chloramphenicol was common (68/109, 62.4% and 56/109, 51.4%, respectively). Fluoroquinolone resistance affected 39.4% of isolates (ciprofloxacin 43/109; levofloxacin 43/109). Resistance to ceftazidime was detected in 34.9% (38/109). By contrast, resistance to piperacillin–tazobactam was rare (8/109, 7.3%), and colistin resistance was observed in 5.5% (6/109). Notably, no resistance was detected against carbapenems (imipenem, meropenem) or against amikacin, ceftazidime–avibactam, ceftolozane–tazobactam or tigecycline.

### Population structure and chromosomal-versus-plasmid distribution of acquired resistance in ESBL-producing *E. coli* from healthy unweaned calves

#### Polyclonal calf ESBL-*E. coli* dominated by phylogroups A and B1

**Fig. 1.**
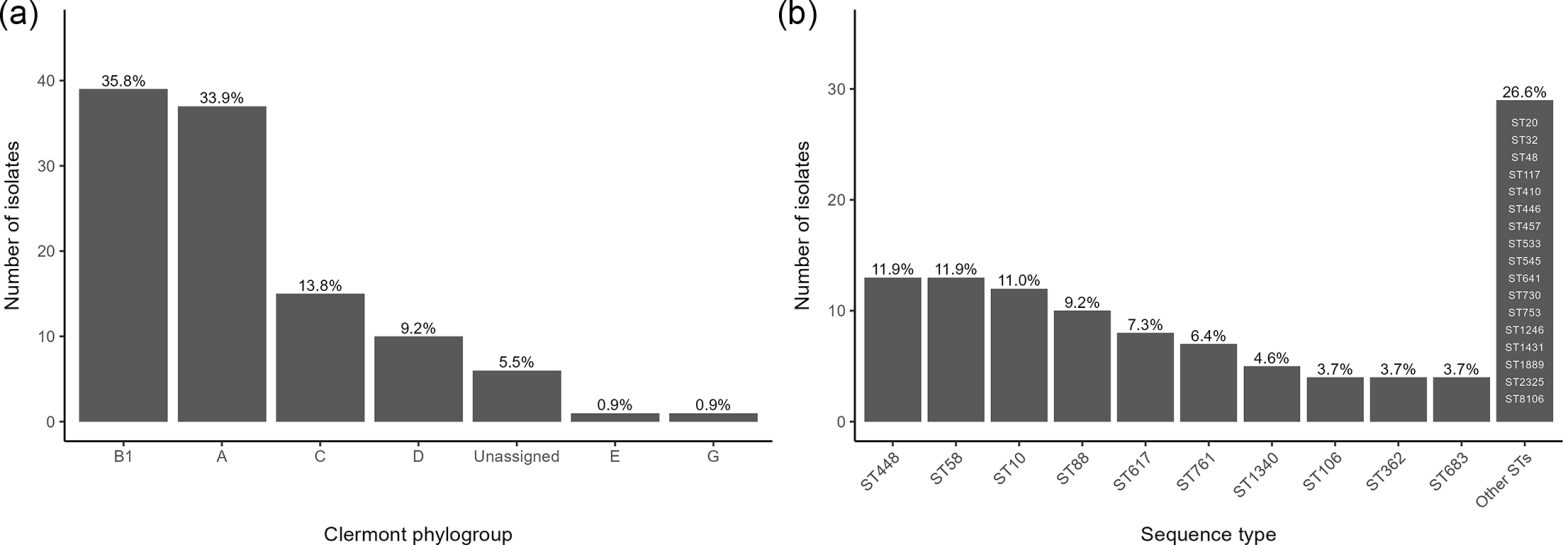
Lineage structure of ESBL-producing *E. coli* (*n*=109) from healthy unweaned dairy calves (*n*=484). (**a**) Clermont phylogroup distribution among 109 calf isolates. (**b**) Multilocus ST distribution. STs with at least four isolates are shown individually, while less common STs are grouped as ‘Other STs’. Bar labels indicate the percentage of isolates in each category.

The ESBL-producing calf isolates formed a polyclonal population dominated by phylogroups B1 (36%) and A (34%) (Fig. 1a). Twenty-eight STs were identified, with ST448 and ST58 being the most frequent (each 12%), confirming substantial clonal diversity (Fig. 1b). Only 6 out of 109 isolates (5.5%) fulfilled molecular criteria for established diarrhoeagenic pathotypes (aEPEC or STEC) and were scattered across diverse phylogenetic backgrounds without dominant clustering, including aEPEC B1/ST20 (*n*=1), STEC-aEPEC D/ST32 (*n*=1), STEC B1/novel ST (*n*=1) and aEPEC A/ST1889 (*n*=3).

### Acquired resistance is predominantly plasmid-borne

Across the calf ESBL population, 54 distinct acquired ARGs were detected. At the gene level, the largest fraction of resistance determinants occurred in both chromosomal and plasmid contexts (44%), whereas others were restricted to plasmids (39%) or chromosomes (17%). Fifteen distinct *β*-lactamase enzyme alleles were identified (CTX-M, SHV, TEM, OXA families). Several major ESBL variants (e.g. *bla*_CTX-M-1_, *bla*_CTX-M-14_ and *bla*_CTX-M-15_) were detected in both chromosomal and plasmid contexts, whereas others (e.g. *bla*_CTX-M-32_, *bla*_CTX-M-55_ and *bla*_CTX-M-65_) were plasmid-exclusive. No plasmid-mediated AmpC genes were identified.

**Fig. 2.**
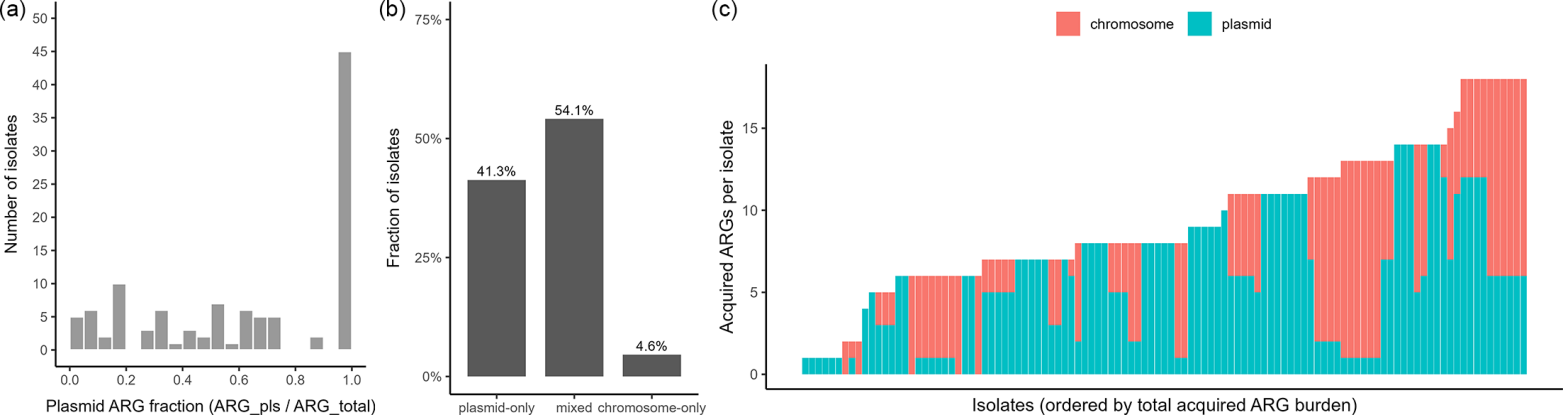
Chromosomal and plasmid distribution of acquired antimicrobial resistance genes (ARGs) in ESBL-producing *E. coli* (*n*=109) from healthy unweaned dairy calves (*n*=484). (**a**) Distribution of the plasmid ARG fraction per isolate (ARG_pls/ARG_total), shown as a histogram to illustrate the extent of plasmid-dominated acquired resistance across the dataset. (**b**) Isolate-level distribution of acquired resistance patterns, classified as plasmid-only, mixed (chromosome+plasmid) or chromosome-only according to the genomic location of acquired ARGs. Percentages indicate the fraction of isolates in each category. (**c**) Number of acquired ARGs per isolate, shown as stacked bars partitioning plasmid-borne (green) and chromosomal (purple) ARG counts; each bar represents one isolate, and isolates are ordered by total number of acquired ARGs.

At the isolate level, resistance was predominantly encoded on plasmids. All isolates carried plasmids (median four per isolate). The median total number of acquired ARGs was eight genes, partitioned into a median of six plasmid-encoded and two chromosomal ARGs. The median plasmid ARG fraction was 0.67 (Fig. 2a). Forty-one per cent of isolates carried exclusively plasmid-borne acquired ARGs, whereas only 4.6% exhibited purely chromosomal acquired resistance. The remaining isolates harboured acquired ARGs in both chromosomal and plasmid contexts (Fig. 2b, c).

### Resistance partitioning is lineage-structured across phylogroups and STs

**Fig. 3.**
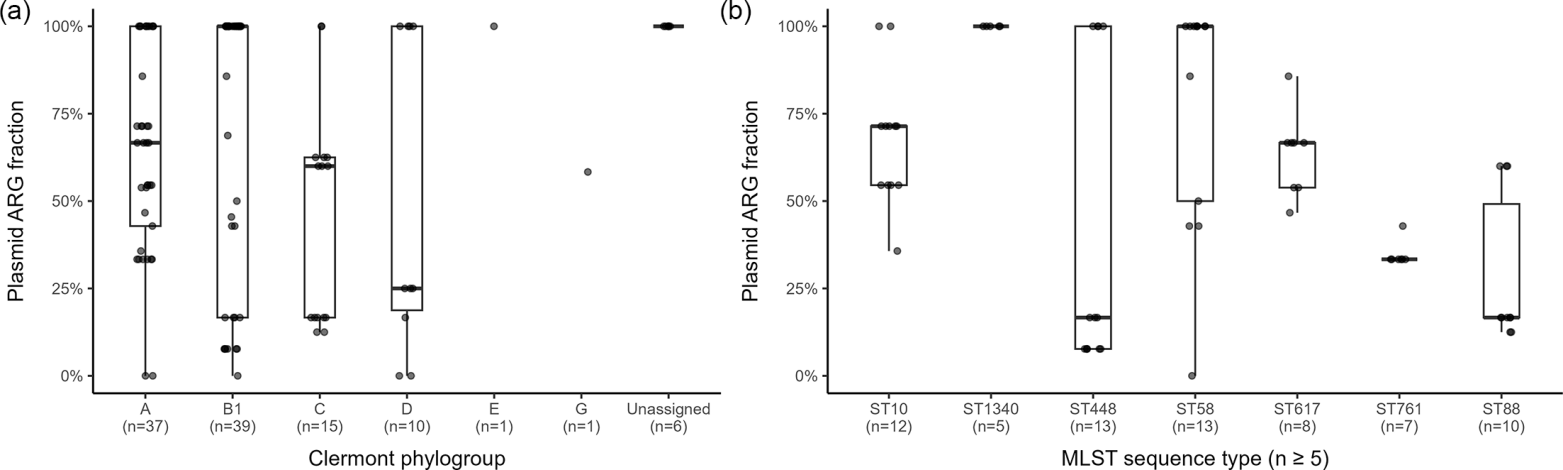
Lineage-dependent plasmid dominance of acquired ARGs in calf ESBL-*E. coli* (*n*=109). (**a**) Plasmid fraction of acquired ARGs per isolate (ARG_pls/ARG_total) stratified by Clermont phylogroup (n as indicated). Plasmid ARG fraction differed significantly between phylogroups (Kruskal–Wallis, *P*=0.01184). (**b**) Plasmid fraction of acquired ARGs stratified by MLST STs represented by ≥5 isolates (n as indicated). Plasmid ARG fraction differed significantly between STs (Kruskal–Wallis, *P*=7.70×10⁻⁵). Boxplots show the median (centre line) and IQR (box); whiskers extend to 1.5×IQR; points represent individual isolates.

The partitioning of acquired resistance between chromosomes and plasmids differed significantly across phylogenetic groups. Specifically, plasmid ARG fraction and chromosomal ARG counts differed between Clermont phylogroups (*P*<0.05), whereas total ARG counts did not. Phylogroup B1 displayed a strongly plasmid-dominated pattern, with a median of zero chromosomal ARGs, whereas phylogroup D showed higher chromosomal contributions, with a median of 4.5 chromosomal ARGs, and a lower plasmid fraction. Phylogroup A exhibited an intermediate configuration (Fig. 3a).

This lineage dependence became more pronounced at the ST level. Among STs represented by ≥5 isolates, the total number of acquired ARGs, as well as chromosomal and plasmid partitions, differed significantly (all *P*<0.01). Certain STs (e.g. ST58 and ST1340) exhibited near-complete plasmid encoding of acquired resistance, whereas others (e.g. ST448 and ST88) showed comparatively greater chromosomal contributions (Fig. 3b).

### Plasmid-encoded virulence is structured and inversely related to plasmid ARG load

VFs were abundant and predominantly chromosomal across all lineages, with median chromosomal VF counts ranging from 180 to 219 genes per isolate. Plasmid-associated VF gene counts were consistently lower, with median values ranging from 11 to 21 genes depending on phylogroup, but functionally structured.

Mobile virulence repertoires were dominated by iron acquisition systems, including the aerobactin operon (*iucABCD*/*iutA*), the salmochelin system (*iroBCDEN*) and the Sit transporter (*sitABCD*). Additional plasmid-encoded factors included serum survival genes (*traT*, *iss*), secreted protease *espP*, fimbrial genes (*faeD*/*faeJ*), bacteriocin loci (*cvaC*, *mchF*) and stability modules such as *ccdB* and integrase-associated elements. Notably, 49 virulence-associated markers were detected exclusively on plasmids and were absent from the chromosomal dataset. These plasmid-exclusive markers included several bacteriocin-associated genes, most prominently *ce1a* (*n*=45), *cia* (*n*=21) and *cvaC* (*n*=17), alongside *ccdB* (*n*=20), the salmochelin locus *iroBCDEN* (each *n*=17) and *traJ* (*n*=17). Additional plasmid-restricted determinants comprised further bacteriocin-associated genes (*cib*, *cma*, *cba*, *cda *and *cei*), toxin-associated loci (*hlyABCD*, *cdt-IIIA/B/C*, *cnf2*, *estIa* and *toxB*), secretion- or colonization-associated genes (*aaiA–P* and *faeE*) and the oxidative stress-associated *katP* gene.

The number of VF genes differed significantly across phylogroups (*P*=0.014), with higher medians in phylogroups C and D compared to A and B1. When stratified by the genomic distribution of acquired ARGs, isolates with exclusively or predominantly chromosomally acquired resistance showed slightly higher numbers of VF genes than isolates with plasmid-only acquired resistance. Correlation analyses demonstrated only weak coupling between virulence and resistance. The total number of VF genes correlated positively with the total number of acquired ARGs (ρ=0.21), but negatively with the number of plasmid-borne ARGs and plasmid ARG fraction (ρ≈−0.2). Thus, stronger plasmid-driven resistance accumulation was associated with modest reductions in overall virulence gene content.

### Chromosomal resistance mutations are pervasive and partially trade off with plasmid dominance

Beyond acquired ARGs, chromosomal resistance-associated gene alterations were highly prevalent (94.5% of isolates), with a median of three mutations per genome. Mutations predominantly affected quinolone resistance–determining regions (*gyrA*, *parC* and *parE*) but also included alterations associated with fosfomycin (*glpT*, *uhpT*), nitrofuran (*nfsA*), colistin (*pmrB*), *β*-lactam regulation (*bla*_TEM_ promoter), outer membrane permeability (*ompF*) and global regulatory pathways (*cyaA*). Mutational count correlated positively with total acquired ARG counts (ρ=0.23), indicating that isolates with expanded acquired resistomes also tended to accumulate chromosomal resistance modifications. Conversely, mutation counts correlated negatively with plasmid ARG fraction (ρ=−0.20), suggesting a partial trade-off between plasmid-dominated resistance and chromosomal adaptation.

### Plasmidome organization, mobility class and co-selection drivers of resistance in ESBL-producing *E. coli* from healthy unweaned calves

#### Conjugative plasmids dominate ARG carriage

**Fig. 4.**
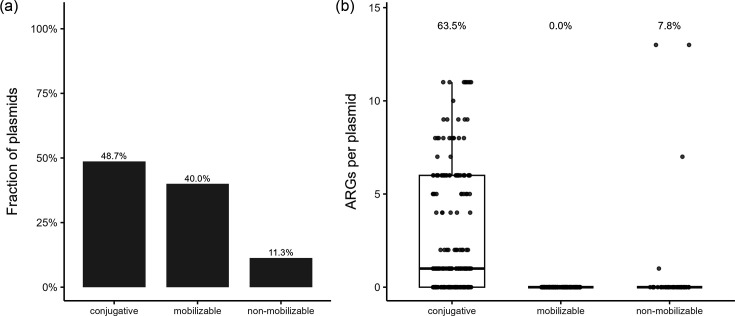
Mobility structure and ARG counts in the calf ESBL *E. coli* plasmidome. (**a**) Distribution of predicted plasmid mobility classes across all calf plasmids (*n*=450). Bars show the fraction of plasmids in each mobility class. (**b**) Distribution of acquired ARGs per plasmid stratified by predicted mobility class (conjugative, mobilizable and non-mobilizable). Points represent individual plasmids; boxplots indicate median and IQR. Percentages above boxes denote the fraction of ARG-positive plasmids within each mobility class.

Predicted plasmid mobility classes were dominated by conjugative and mobilizable plasmids, which represented 48.7 and 40.0% of all calf plasmids, respectively, while non-mobilizable plasmids accounted for 11.3% (Fig. 4a). Across 450 calf plasmids, ARG carriage was strongly mobility-dependent: conjugative plasmids (*n*=219) accounted for 600 of 634 plasmid-borne ARG occurrences (94.6%), whereas non-mobilizable plasmids (*n*=51) contributed only 34 (5.4%). Accordingly, 63.5% of conjugative plasmids were ARG-positive (139 out of 219), compared with only 7.8% of non-mobilizable plasmids (4 out of 51). At the per-plasmid level, conjugative plasmids carried substantially higher ARG counts (median 1; mean 2.74; range 0–11) than mobilizable plasmids (median 0; mean 0.15). This difference was highly significant (Kruskal–Wallis χ²=185.15, *P*=3.64×10⁻⁴²; Wilcoxon BH-adjusted *P*=3.66×10⁻⁴²) (Fig. 4b).

### IncF backbones and recurrent cluster hubs concentrate resistance

ARG carriage was highly concentrated within specific plasmid backbones. Of 634 total ARG occurrences (mean 1.41 per plasmid), 63.0% were embedded within IncF-type plasmids. Overall, IncF-type plasmids represented 62.2% of all plasmids (280 out of 450), with IncFIA (*n*=99), IncFIB (*n*=96) and IncFIC (*n*=68) being the most frequent subtypes. IncFIA, IncFIB and IncFIC contributed 23.0%, 20.2% and 16.0% of total plasmid-associated ARG occurrences, respectively, with the remainder contributed by other IncF subtypes. While IncFIA and IncFIB plasmids showed modest median ARG loads (median 1), IncFIC plasmids were more heavily loaded (median 4; maximum 11). Other replicon types contributed only minor fractions of the total ARG signal.

**Fig. 5.**
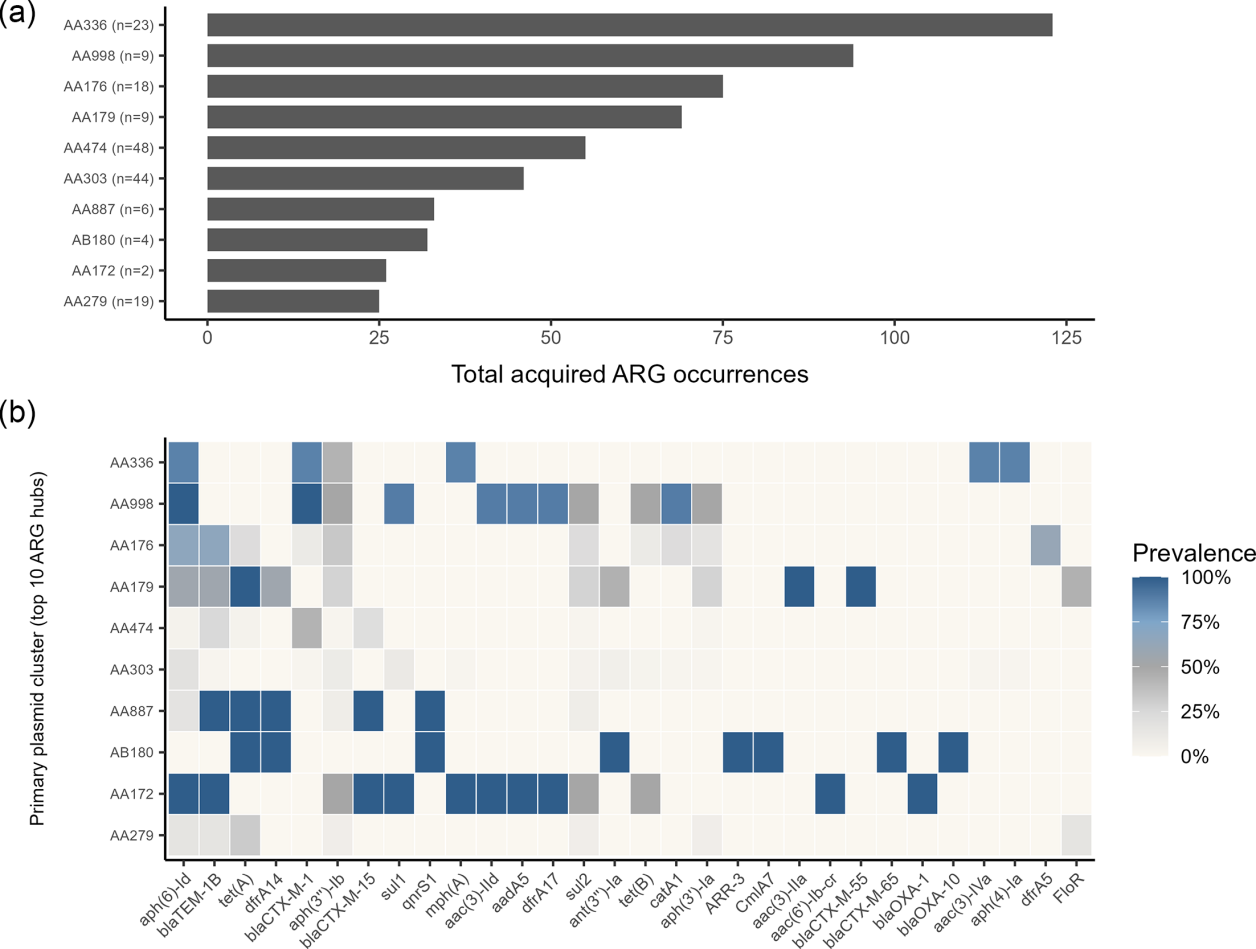
Backbone hubs and ARG signatures in the calf ESBL *E. coli* plasmidome. (**a**) ARG hub structure across primary plasmid clusters. Bars show the total number of acquired ARG occurrences summed across plasmids within each of the top 10 ARG-carrying primary clusters; cluster labels indicate the number of plasmids assigned to each cluster (**n**). (**b**) ARG composition of major hub clusters. Heatmap depicts within-cluster prevalence (0–100%) of the top 30 most recurrent ARGs across the top 10 ARG hub clusters (rows), highlighting cluster-specific resistance ‘signatures’ and shared multidrug modules.

Stratification by primary plasmid cluster revealed an even sharper hub structure. The four most ARG-rich clusters (AA336, AA998, AA176 and AA179) accounted for 56.9% of the total plasmid-associated ARG occurrences (Fig. 5a). Cluster AA336 alone contributed 165 ARG occurrences (26.0%), with 94.1% of plasmids being ARG-positive. Clusters AA176 and AA179 exhibited particularly dense multidrug configurations (medians 7–8 ARGs per plasmid).

Across clusters, resistance signatures were highly recurrent. For example, AA336 was enriched for *sul2* (0.912 prevalence), *aph(6)-Id* (0.824), *aph(3″)-Ib* (0.794), *tet(A*) (0.794) and *bla*_TEM-1B_ (0.706). Cluster AA176 was characterized by co-occurrence of *bla*_CTX-M-1_ with aminoglycoside and sulphonamide resistance determinants, whereas AA179 combined *bla*_TEM-1B_ and *bla*_CTX-M-15_ with *sul2* and *tet(A*), reflecting structured multidrug resistance modules (Fig. 5b).

Altogether, 91.2% of plasmid-associated ARG occurrences were concentrated within the ten most ARG-rich clusters, indicating that resistance dissemination is dominated by a limited, recurrent backbone repertoire.

### ARG–BMRG coupling reflects shared mobile modules

ARGs and BMRGs were unevenly distributed across plasmids. The median numbers of ARGs and BMRGs were both zero per plasmid, indicating that these genes were concentrated in a subset of plasmid backbones. Overall, 31.8% of plasmids carried ≥1 ARG and 39.3% carried ≥1 BMRG.

**Fig. 6.**
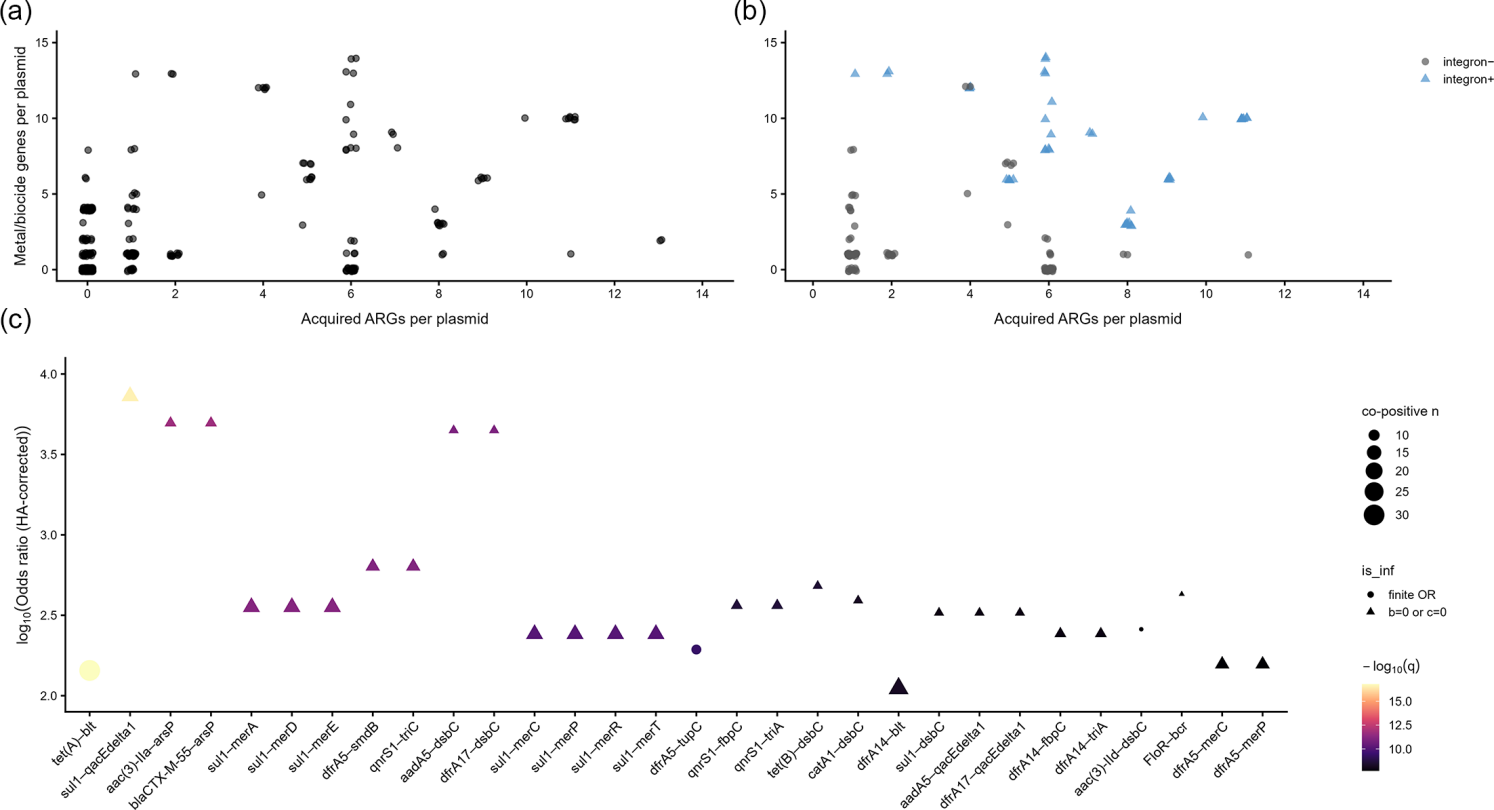
Co-selection of acquired ARGs, as well as BMRGs on calf *E. coli* plasmids. (**a**) Relationship between the number of acquired ARGs per plasmid and the number of BMRGs per plasmid across all plasmids. Points show individual plasmids with jitter applied for readability. (**b**) Same analysis restricted to ARG-positive conjugative plasmids; class 1 integron-positive plasmids are highlighted. (**c**) Top enriched ARG–BMRG co-occurrence pairs among ARG-positive conjugative plasmids. Enrichment was tested using Fisher’s exact test for each ARG–BMRG pair, and multiple testing was controlled using BH false discovery rate (**q**). Point size indicates the number of co-positive plasmids (**n**). Colour encodes statistical support as −log10(q). The x-axis shows the log10-transformed OR calculated with Haldane–Anscombe correction (0.5 continuity correction) to yield finite estimates; triangles mark pairs with complete separation (b=0 or c=0).

ARG and BMRG counts were strongly correlated at the plasmid level (Spearman ρ=0.574, *P*=8.0×10⁻⁴¹) (Fig. 6a). Among ARG-negative plasmids, only 20.5% carried BMRG determinants, compared with 79.7% of ARG-positive plasmids (OR=15.10, 95% CI 9.07–25.82, *P*=1.41×10⁻³³). Within ARG-positive conjugative plasmids (*n*=139), ARG load (median 5; IQR 4–9) correlated positively with the number of BMRGs (ρ=0.393, *P*=1.37×10⁻⁶), indicating quantitative co-accumulation within transferable backbones (Fig. 6b).

Gene-level co-occurrence analyses revealed structured linkage patterns consistent with shared mobile modules (Fig. 6c). The strongest signal was *tet(A)–blt* (OR=205.35, q=6.29×10⁻¹⁷), linking tetracycline resistance with QAC-associated efflux. Integron-associated configurations were prominent: *sul1* co-occurred perfectly with *qacEdelta1* (OR → ∞, q=1.10×10⁻¹⁶) and with *mer* operon genes (*merADE*). Additional high-confidence pairings included *bla*_CTX-M-55_–*arsP* and *aac(3)-IIa–arsP* (each OR → ∞), as well as *qnrS1* associations with triclosan-related efflux loci (*triC*, *triA*). These patterns indicate non-random integration of antibiotic and metal/biocide resistance modules within specific plasmid backbones.

### Class 1 integrons define conjugative multidrug platforms

**Fig. 7.**
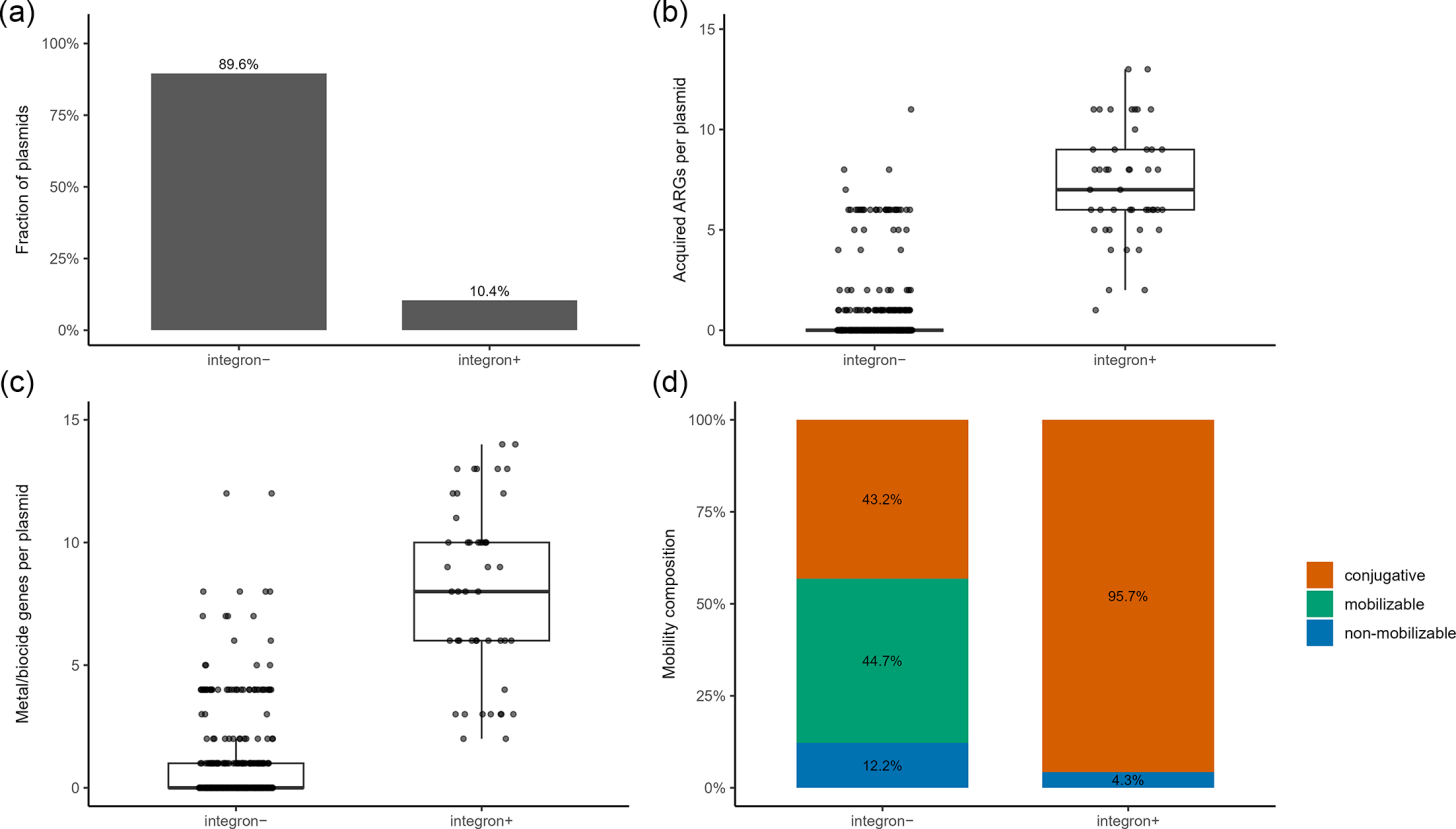
Class 1 integrons define rare but highly enriched multidrug resistance platforms in the calf plasmidome. (**a**) Fraction of plasmids classified as integron-negative (integron−) or integron-positive (integron+; 10.4% of all plasmids; *n*=450). (**b**) Number of acquired ARGs per plasmid stratified by integron status. Integron+ plasmids carried significantly more ARGs than integron− plasmids (Wilcoxon rank-sum: W=633.5, *P*<2.2×10⁻¹⁶). (**c**) BMRG numbers per plasmid stratified by integron status. Integron+ plasmids carried significantly more BMRG determinants than integron− plasmids (Wilcoxon rank-sum: W=678, *P*<2.2×10⁻¹⁶). Boxplots show median and IQR; whiskers indicate 1.5×IQR; points represent individual plasmids. (**d**) Predicted mobility composition within integron− and integron+ plasmids.

Class 1 integrons (*intI1*-positive) were present in 47 out of 450 plasmids (10.4%) (Fig. 7a). Despite their minority status, integron-positive plasmids carried substantially higher ARG counts. Integron-positive plasmids harboured a median of eight ARGs (mean 7.44), compared with zero among integron-negative plasmids (mean 0.77) (Wilcoxon *P*=6.22×10⁻³⁴) (Fig. 7b). Their ARG repertoires were dominated by aminoglycoside [*aph(6)-Id*, *aph(3'')-Ib *and *aph(3')-Ia*], sulphonamide (*sul1*, *sul2*), trimethoprim (*dfrA14*) and tetracycline [*tet(A)*] resistance determinants, with recurrent *β*-lactam resistance genes, including *bla*_TEM-1B_ and the ESBL genes *bla*_CTX-M-1_ and *bla*_CTX-M-15_.

At the binary level, integron carriage was almost deterministic for ARG positivity (OR → ∞, *P*=2.68×10⁻²⁴). A parallel enrichment was observed for metal/biocide determinants: integron-positive plasmids carried a median of nine BMRGs (Wilcoxon *P*=3.00×10⁻²⁹) and showed strong binary enrichment (OR → ∞, *P*=1.14×10⁻¹⁹) (Fig. 7c). Integrons were almost exclusively associated with conjugative plasmids (45/47; 95.7%), underscoring their embedding within transferable multidrug platforms (Fig. 7d).

### Host-associated differences are lineage-structured

#### Unadjusted host comparisons suggest differences in resistance partitioning

Direct comparison of calf (*n*=109) and human (*n*=479) isolates revealed marked differences in the genomic distribution of acquired resistance. Plasmid carriage was universal in calves (median four plasmids per isolate), whereas only 80.6% of human isolates carried plasmids (median two), indicating a lower plasmid load in the human reference dataset. Calf isolates exhibited higher total numbers of acquired ARGs (median eight vs six; *P*=1.06×10⁻⁶), driven by both chromosomal (median two vs zero; *P*=1.02×10⁻⁴) and plasmid-borne ARG counts (median six vs three; *P*=0.0011). Despite the higher absolute number of acquired ARGs in calves, the relative distribution of acquired resistance differed between hosts: the plasmid ARG fraction was higher in human isolates (median 0.769 vs 0.667; *P*=0.0032), indicating a stronger plasmid contribution to acquired resistance in the human dataset. The distribution of isolate-level categories (plasmid-only, mixed and chromosome-only) also differed significantly between hosts (χ²=20.14, *P*=4.24×10⁻⁵). Because the two datasets differed substantially in population structure, these host-level comparisons were re-evaluated after stratification by phylogroup and ST.

### Lineage-aware analyses localize isolate-level host effects to phylogroup C

To disentangle host-associated signals from clonal structure, isolate-level analyses were repeated after restricting to plasmid-positive isolates and explicitly controlling for lineage. In negative binomial models, including ST as a fixed effect, host origin was no longer associated with the number of plasmid-borne ARGs (IRR 1.11; *P*=0.505). ST-matched comparisons across lineages shared between hosts likewise showed no significant host effect (paired Wilcoxon test on per-ST medians, *P*=0.572). Thus, when equivalent clonal backgrounds were compared, overall plasmid ARG load did not systematically differ between calf and human isolates. However, phylogroup-stratified analyses revealed a lineage-specific amplification pattern. Within phylogroup C, human isolates carried substantially higher numbers of plasmid-borne ARGs than calf isolates (median 11 vs 3; BH-adjusted *P*=4.8×10⁻⁵). Phylogroup C comprised partially overlapping ST compositions between hosts, including shared lineages (ST410 and ST88), as well as host-specific STs (humans: ST90, ST23, ST2851; calves: ST8106). Thus, the observed amplification in humans cannot be attributed to a single expanding clone but reflects host-associated differences within a shared phylogenetic background. Interaction modelling yielded an IRR of 2.97 (95% CI 1.64–5.36) for the human effect within phylogroup C, whereas no significant host effects were observed in phylogroups A, B1 or D.

### Human plasmidomes exhibit lineage-adjusted ARG loading beyond conjugative backbones

**Fig. 8.**
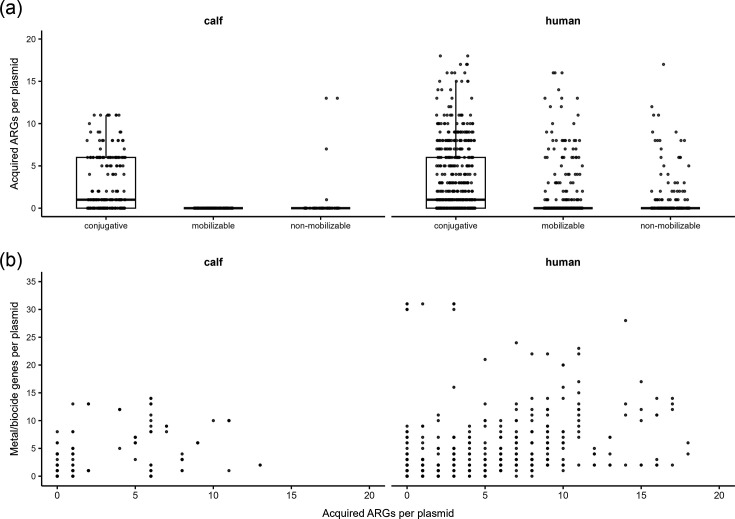
Host-associated differences in plasmid mobility, ARG counts and co-selection with metal/biocide resistance genes. (**a**) Distribution of acquired ARGs per plasmid across mobility classes (conjugative, mobilizable and non-mobilizable) in calves (*n*=450) and humans (*n*=1,163). Boxes show IQR with median; individual plasmids are shown as dots. (**b**) Correlation between ARG and BMRG loads per plasmid in calves and humans. Each dot represents one plasmid. Spearman correlation: calves ρ=0.574 (*P*<2.2×10⁻¹⁶); humans ρ=0.733 (*P*<2.2×10⁻¹⁶).

We next assessed whether host-associated differences persist at the plasmid level after accounting for lineage structure. Plasmid mobility composition differed between datasets (χ² = 11.10, *P*=0.0039). Conjugative plasmids constituted 48.7% of calf plasmids (219 out of 450) and 41.8% of human plasmids (486 out of 1,163), whereas non-mobilizable plasmids were proportionally more frequent in human-derived isolates (17.4% vs 11.3%). Negative binomial regression, including host, mobility class and phylogroup, demonstrated that human plasmids carried significantly higher ARG counts after adjustment (IRR 1.66, 95% CI 1.21–2.27; *P*=0.0017). As expected, mobilizable plasmids harboured markedly fewer ARGs than conjugative plasmids in the calf background (IRR 0.04; *P*<10⁻³⁵). However, a highly significant host×mobility interaction (IRR 4.61, 95% CI 2.66–8.00; *P*=4.9×10⁻⁸) indicated that mobilizable plasmids in the human dataset accumulated ARGs to a substantially greater extent than mobilizable plasmids in calves. Logistic models of ARG positivity confirmed this interaction (OR 8.63; *P*=7.8×10⁻⁵) (Fig. 8a).

### Lineage-adjusted amplification of transferable ARG–BMRG co-selection and integron enrichment in the human plasmidome

Quantitative coupling between acquired ARGs and BMRGs was observed in both hosts but was substantially stronger in human-derived isolates. Across all plasmids, ARG–BMRG counts correlated positively in calves (ρ=0.571, *P*<10⁻³⁸) and more strongly in human-associated isolates (ρ=0.733, *P*<10⁻¹⁹⁵) (Fig. 8b). Binary enrichment of BMRGs among ARG-positive plasmids was likewise stronger in human-associated isolates (OR=28.81 vs 15.10).

Phylogroup-stratified analyses showed that enhanced coupling in human-associated isolates was not confined to a single lineage. Although phylogroup C exhibited particularly strong correlation in humans (ρ=0.776 vs 0.397 in calves), elevated coupling was observed across multiple phylogroups. Importantly, restriction to conjugative plasmids amplified host divergence: ARG–BMRG coupling within conjugative plasmids was weak in calves (ρ=0.207, *P*=0.002) but strong in humans (ρ=0.543, *P*<10⁻³⁸).

Gene-level co-occurrence patterns reflected these structural differences. In human isolates, extreme enrichment of *sul1–qacEdelta1* (OR=8,396) indicated extensive integron-linked QAC modules. Additional enriched pairings, i.e. *tet(A)–blt*, *mph(A)–qacEdelta1*, *floR–bcr*, *bla*_NDM-5_–*qacEdelta1* and *mcr-1.1–pmrC*, demonstrated recurrent integration of disinfectant and metal resistance into multidrug plasmids. In calves, significant ARG–BMRG linkages were detectable but largely confined to specific ARG-rich plasmid clusters rather than broadly embedded across the transferable plasmid pool.

Class 1 integrons provided a structural explanation for this divergence. Integron-positive plasmids were nearly twice as frequent in humans (19.8%) as in calves (10.4%). In both hosts, integron-positive plasmids exhibited substantially higher ARG counts and near-complete separation for ARG positivity (OR → ∞), as well as strong BMRG enrichment (OR=83.76 in humans). Integrons were predominantly associated with conjugative plasmids, linking multidrug resistance and metal/biocide modules to transferable backbones.

### Virulence content is decoupled from plasmid-mediated resistance across hosts

The total number of VF genes was comparable between hosts (median 213 in calves vs 202 in humans), and chromosomal VF content was nearly identical (median 196 in both datasets), indicating a largely conserved core virulence repertoire. However, plasmid-associated VF repertoires differed substantially. Calf isolates carried more plasmid-encoded VF genes (median 17 vs 2), whereas human plasmid VF content was more limited and enriched for stabilization and fitness-associated loci. In both hosts, the total number of VF genes was negatively correlated with plasmid ARG load (calves ρ=−0.251; humans ρ=−0.326), indicating partial decoupling of virulence content from plasmid-mediated resistance accumulation.

In the human dataset, mobile VF repertoires were dominated by *traT*, integrase-associated loci, *ccdB*, iron acquisition systems (*sitABCD*, *iucABCD*/*iutA *and *iroBCDEN*), serum survival determinants (*iss2*) and haemolysin transport-associated loci (*hlyB*), consistent with plasmid stabilization and host adaptation functions rather than classical virulence expansion.

## Discussion

### Plasmid-dominated acquired resistance in calf ESBL-producing *E. coli* is structured by phylogroup and ST

In calf ESBL-producing *E. coli*, acquired resistance was largely maintained on plasmids, supporting a dominant role of mobile genetic elements in livestock-associated ESBL resistomes [10,30]. The recurrence of the same ARG variants in chromosomal and plasmid contexts further suggests ongoing exchange between mobile and chromosomal reservoirs through transposition and recombination [31,32]. This plasmid dominance was not uniform but varied across phylogroups and STs, consistent with lineage-specific differences in plasmid compatibility, restriction–modification barriers, recombination dynamics and ecological history [33,35]. Thus, the calf ESBL resistome should be considered as a lineage-structured mosaic of resistance stabilization strategies rather than a single generalizable pattern.

### Virulence and chromosomal mutations indicate parallel adaptation routes

In contrast to acquired ARGs, virulence genes remained largely chromosomal and phylogenetically structured, consistent with conserved lineage-linked virulence repertoires in *E. coli* [36,38]. The weak negative association between plasmid-borne ARGs and total VF content argues against a simple virulence–resistance trade-off but supports partial decoupling between resistance-focused plasmidomes and broader virulence expansion. Calf plasmids nevertheless carried a focused accessory repertoire enriched for iron acquisition, serum resistance, adhesin-associated loci, bacteriocin genes and maintenance functions, more consistent with colonization fitness and persistence than with broad virulence amplification [8,39, 40]. Together with the widespread chromosomal resistance mutations, these patterns point to parallel evolutionary routes in which lineages differ in the relative contribution of plasmid-mediated acquisition versus chromosome-centred adaptation [9,41,45].

### In calves, resistance dissemination is concentrated in conjugative IncF hubs amplified by integrons

At the plasmidome level, resistance dissemination was concentrated in conjugative IncF backbones and a limited number of recurrent plasmid clusters, indicating that spread in calves is driven by a small set of successful transferable backbones rather than diffuse sharing across plasmid diversity [15,30, 46,48]. Co-localization of ARGs with BMRGs, including recurrent *sul1–qacEΔ1* and *mer/ars*-linked configurations, further supports mixed-selective maintenance of these multidrug plasmids under antibiotic, disinfectant and metal exposure [7,49,52]. In dairy production, these co-selection patterns likely reflect farm-system exposure rather than direct antimicrobial treatment of calves alone. Antimicrobials are used only selectively in neonatal calf diarrhoea and, when used, typically include aminopenicillins or potentiated sulphonamides, whereas fluoroquinolones are regarded as last-choice agents [53,54]. By contrast, mastitis management and dry-off therapy represent major antimicrobial use points in dairy herds, with intramammary dry-cow treatments commonly involving *β*-lactams such as cloxacillin, cephalonium or cefquinome [55,56]. The resistance profiles observed here may therefore reflect maternal and environmental transmission within the farm system, including exposure through colostrum and shared milking or feeding equipment [13,57]. This interpretation is consistent with studies showing ESBL-*E. coli* transmission via colostrum, contamination of milking equipment and frequent bacterial contamination of feeding nipples and suckling buckets, especially under insufficient cleaning and biofilm persistence [58]. The QAC-linked signal may fit this farm-level exposure model, particularly where QAC-based disinfectants are mainly used for milking equipment sanitation and similar cleaning practices extend to calf-feeding equipment [59]. Under such conditions, insufficient rinsing and persistent biofilms on nipples or buckets could help maintain QAC-linked resistance plasmids even in the absence of direct antimicrobial treatment of calves. Class 1 integrons likely intensify this process by amplifying ARG and BMRG accumulation within highly transmissible conjugative backbones [1,60,62].

### Host-context comparison reveals lineage-structured differences but persistent human-specific plasmid remodelling

Cross-host comparison showed that much of the apparent isolate-level host signal was attributable to differences in phylogroup and ST composition, underscoring the need to account for population structure in such analyses [2,3, 63, 64]. The persistence of a signal in phylogroup C nevertheless suggests that host-associated amplification can occur within shared phylogenetic backgrounds rather than arising solely from different clone repertoires [65,67].

By contrast, plasmid-level differences persisted after adjustment: human-associated *E. coli* showed broader redistribution of ARGs across mobility classes, especially mobilizable plasmids, together with stronger and more pervasive ARG–BMRG coupling. This pattern implies a more extensively remodelled plasmidome in which antibiotic, biocide and metal resistance modules are integrated across diverse transferable backgrounds rather than confined to a few recurrent hubs [1,68,70]. The higher frequency of integron-positive plasmids and pronounced *sul1–qacEΔ1* enrichment in humans provide a plausible mechanistic basis for this divergence, consistent with broader embedding of integron-linked QAC-associated modules into multidrug resistance plasmids [71,73].

Virulence patterns reinforced these functional differences: calf isolates carried more plasmid-associated VF genes, whereas human plasmidomes were more focused on persistence- and fitness-associated loci. Together with the negative association between the total number of VF genes and plasmid ARG load in both hosts, this supports partial decoupling between plasmid-mediated resistance accumulation and broader virulence expansion [8,36, 74].

Overall, host-associated differences in *E. coli* resistance are shaped less by gene content alone than by how resistance modules are organized within lineage-specific plasmid backgrounds. In calves, ARGs accumulated in a limited set of conjugative, IncF-enriched backbones, whereas the human plasmidome showed broader mobility-class redistribution and tighter coupling to BMRGs, consistent with host-context-dependent selection on mobile resistance platforms. This distinction is important for AMR ecology because resistance genes located on stable chromosomes, recurrent conjugative plasmids or broadly redistributed mobile elements differ in their persistence, co-selection potential and likelihood of dissemination. Future AMR surveillance and comparative genomics should therefore move beyond resistance gene inventories and explicitly incorporate genomic localization, plasmid mobility, backbone identity and co-localized adaptive traits.

## Supplementary material

10.1099/mgen.0.001783 Table S1.

10.1099/mgen.0.001783 Table S2.

## Abbreviations

AMR: antimicrobial resistance
ARGs: antibiotic resistance genes
BH: Benjamini–Hochberg
BMRGs: biocide/metal resistance genes
ESBL: extended-spectrum *β*-lactamase
IQRs: interquartile ranges
ORs: odds ratios
PA: percentage alignment
ST: sequence type
VFs: virulence factors
